# Allantoic fluid metabolome reveals specific metabolic signatures in chicken lines different for their muscle glycogen content

**DOI:** 10.1038/s41598-023-35652-0

**Published:** 2023-05-31

**Authors:** Angélique Petit, Sophie Tesseraud, Stéphane Beauclercq, Lydie Nadal-Desbarats, Estelle Cailleau-Audouin, Sophie Réhault-Godbert, Cécile Berri, Elisabeth Le Bihan-Duval, Sonia Métayer-Coustard

**Affiliations:** 1 INRAE, Université de Tours, BOA, 37380 Nouzilly, France; 2 INSERM, Université de Tours, iBrain, 37000 Tours, France

**Keywords:** Biochemistry, Physiology

## Abstract

Nutrient availability in eggs can affect early metabolic orientation in birds. In chickens divergently selected on the *Pectoralis major* ultimate pH, a proxy for muscle glycogen stores, characterization of the yolk and amniotic fluid revealed a different nutritional environment. The present study aimed to assess indicators of embryo metabolism in pHu lines (pHu+ and pHu−) using allantoic fluids (compartment storing nitrogenous waste products and metabolites), collected at days 10, 14 and 17 of embryogenesis and characterized by 1H-NMR spectroscopy. Analysis of metabolic profiles revealed a significant stage effect, with an enrichment in metabolites at the end of incubation, and an increase in interindividual variability during development. OPLS-DA analysis discriminated the two lines. The allantoic fluid of pHu− was richer in carbohydrates, intermediates of purine metabolism and derivatives of tryptophan-histidine metabolism, while formate, branched-chain amino acids, Krebs cycle intermediates and metabolites from different catabolic pathways were more abundant in pHu+. In conclusion, the characterization of the main nutrient sources for embryos and now allantoic fluids provided an overview of the *in ovo* nutritional environment of pHu lines. Moreover, this study revealed the establishment, as early as day 10 of embryo development, of specific metabolic signatures in the allantoic fluid of pHu+ and pHu− lines.

## Introduction

During the incubation period (21 days in chickens), the embryo depends essentially on the nutrient content of the egg and its metabolism shifts in accordance with the type of substrate available and oxygen supply^1^. Access to O_2_ fully supports the complete combustion of fatty acids, which are used as the primary source of energy for embryo development^2^. According to Nasir and Peebles^3^, intensive selection for a high growth rate and muscle yield has changed the nutrient requirements of chicken embryos, with a potential imbalance between nutrient requirements and reserves. pHu+ and pHu− lines have been divergently selected based on the ultimate pH (pHu) of the meat, a proxy for muscle glycogen reserves^4^. The effects of this selection on broiler metabolism have been studied at 6 weeks of age through the quantification of muscle and serum metabolites by nuclear magnetic resonance (NMR) spectroscopy^5^. Chickens with low muscle pHu (pHu−) and high glycogen reserves are characterized by an over-representation of carbohydrate-related metabolites in serum and muscle, while chickens with high muscle pHu (pHu +) and low glycogen reserves exhibit markers of oxidative stress, muscle proteolysis, amino acid catabolism and β-oxidation. A liquid chromatography–mass spectrometry approach performed on 17-day-old animals also revealed different blood lipidomic signatures between the two divergent lines^6^. Glycerolipids (involved in energy storage) and ceramides were in higher concentrations in the blood of pHu− birds, whereas glycerophospholipids and sterols were more abundant in pHu+. Finally, it has been shown that differences between lines related to protein and energy metabolism are established from hatching^7^.

To understand the metabolic orientation of embryos and the potential link with *in ovo* nutrient availability, the metabolome of the yolk and amniotic fluid was studied in pHu− and pHu+ lines before incubation (E0) and at day 10 of embryo development (E10)^8^. Metabolomic analysis of these two compartments revealed differences in metabolite composition and abundance in embryonated eggs of pHu+ and pHu− lines, which may explain subsequent metabolic and developmental specificities. To further describe the nutritional environment of the embryos, the present study investigated the metabolic composition of the allantoic fluid of both lines during development. Indeed, the allantoic fluid can store nitrogenous waste products, free amino acids and other important compounds for late embryo nutrition^9–11^.

Allantois of the chick embryo appears at about 3 days of incubation as an evagination of the gut wall. Soon after its appearance, a branched network of capillaries is formed. It receives blood via the allantoic arteries and returns it to the body via the allantoic veins. Several studies revealed that the blood, allantoic and amniotic fluid of chicken embryos, which are separated from each other by complex and specific barriers, contain many amino acids and related compounds^12,13^. According to Da silva et al.^10^, some nutrients, such as proteins and peptides, secreted into the allantoic fluid can be digested to provide free amino acids, which can be reabsorbed by the allantoic membrane and redirected to the embryo via the chorioallantoic capillary plexus. Since the allantoic fluid pH decreases in the last stages of development to reach intestinal pH values, optimal conditions can be found in the allantois for protein pre-digestion (activation of proteases).

To assess the developmental patterns of pHu+ and pHu− metabolism during embryogenesis, a metabolomic analysis by ^1^H-NMR spectroscopy was conducted on allantoic fluids at E10, E14 and E17. These stages were chosen according to the availability of substrates in the eggs and the literature on embryo development and metabolism^1,2,14–16^. At E10, yolk lipids are used as the main energy substrate. The stage E14 is characterized by the complete differentiation of the chorioallantoic membrane, an intensification of nutrient transfers to the embryonic tissues (e.g., lipids), the opening of the sero-amniotic connection allowing the albumen to enter the amniotic cavity and by a rapid growth of the embryos. At E17, nutrient sources diversify, the growth rate decreases and the embryo prepares to hatch. The objective was to obtain indirect indicators from the metabolic status of the embryos and to determine the stage at which metabolic orientation was established in the two divergent lines.

## Results

### Description of the metabolomes obtained at E10, E14 and E17

**Figure 1:**
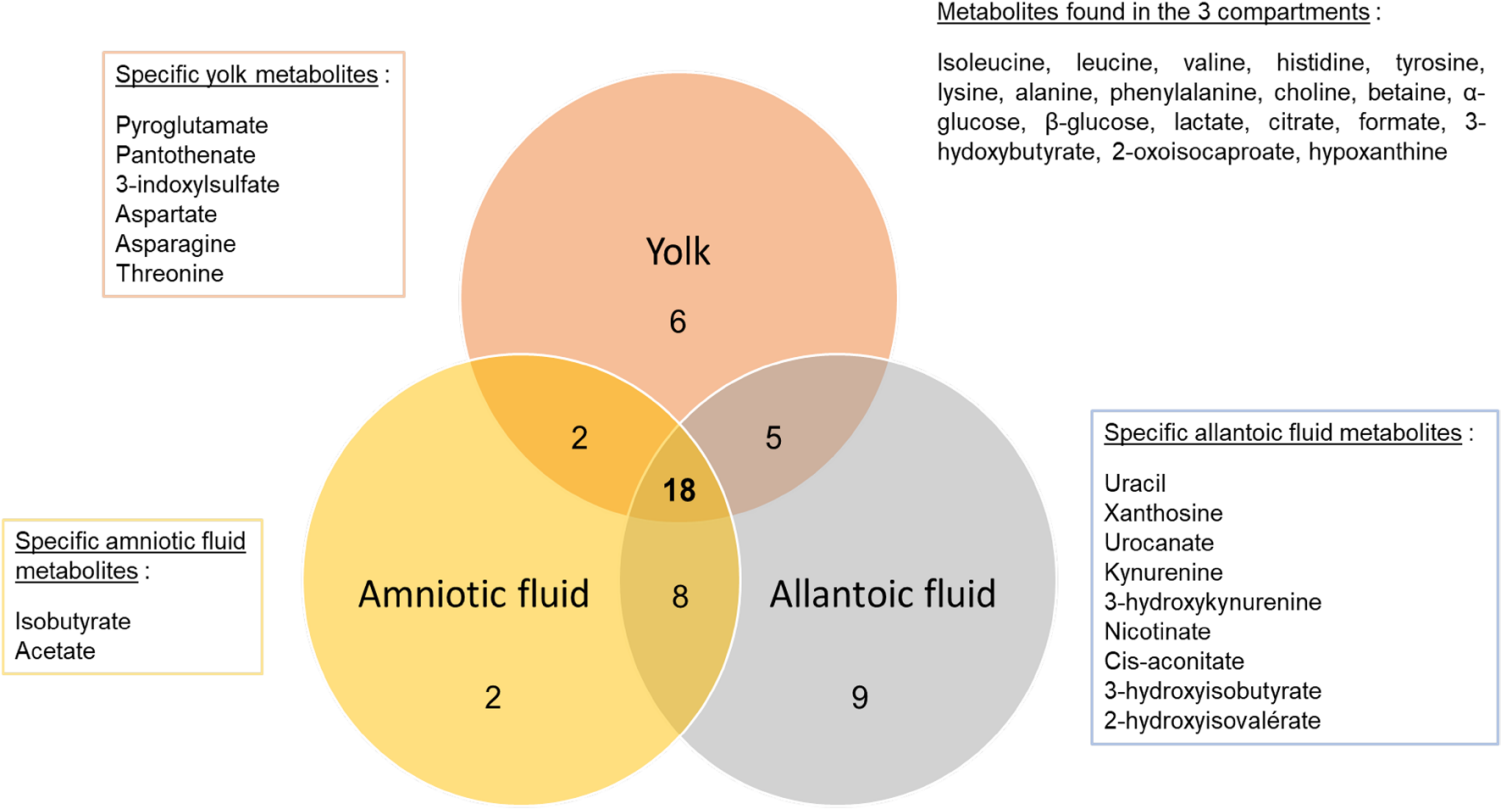
Venn diagram representing metabolites present in allantoic fluid (present study), yolk and amniotic fluid^8^. It compiles metabolites analyzed on the 10th day of incubation (E10) in the amniotic fluid, E0 (before incubation) and E10 in the yolk, and E10, E14 and E17 in the allantoic fluid. Metabolic profile analysis identified 31 metabolites in yolk, 30 in amniotic fluid and 40 in allantoic fluid referenced in the databases. Of the 40 metabolites identified in the allantoic fluid, 18 were common to all 3 compartments, 5 were present in both yolk and allantoic fluid, and 8 were present in both amniotic fluid and allantoic fluid. Specific metabolites of the yolk (n = 6), amniotic fluid (n = 2) and allantoic fluid (n = 9) are specified in the colored boxes (in orange, yellow and gray, respectively).

To characterize the metabolic profiles of pHu− and pHu+ lines and their evolution during embryogenesis, we analyzed the allantoic fluid by ^1^H-NMR spectroscopy at E10, E14 and E17. Figure 1 summarizes the metabolites found in yolk, amniotic fluid and allantoic fluid^8^. Forty distinct metabolites were identified in the allantoic fluid of the pHu lines (Table 1; representative NMR spectra in supplementary Fig. 1). Among these 40 metabolites, 18 were common to all 3 compartments, 5 were present in both yolk and allantoic fluid (glutamate, glycine, glucose, mannose, fumarate), and 8 were present in both amniotic fluid and allantoic fluid (creatine, inosine, cytidine, dimethylamine, dimethylsulfone, pyruvate, 2-hydroxy(HO)butyrate, 3-methyl-2-oxovalerate). Nine metabolites were found only in the allantoic fluid (uracil, xanthosine, urocanate, kynurenine, 3-HOkynurenine, nicotinate, cis-aconitate, 3-HOisobutyrate, 2-HOisovalerate). Pyroglutamate, pantothenate, 3-indoxylsulfate, aspartate, asparagine and threonine were specific to the yolk while isobutyrate and acetate were specific to amniotic fluid.

**Table 1:** List of metabolites identified at E10, E14 and E17 in the allantoic fluid of pHu+ eggs.

Classes	Metabolites pHu+	E10	E14	E17	Fold change E17/E10	Fold change E17/E14
Amino acids, peptides, and analogues	Alanine-148	0.282 ± 0.019^b^	0.216 ± 0.022^b^	0.723 ± 0.047^a^	2.6	3.3
	Betaine-327	2.222 ± 0.088^b^	4.568 ± 0.294^b^	17.029 ± 3.618^a^	**7.7**	3.7
	Betaine-390	0.606 ± 0.023^b^	1.092 ± 0.061^b^	4.528 ± 0.779^a^	**7.5**	4.1
	Creatine-304	0.417 ± 0.011^c^	0.946 ± 0.079^b^	3.478 ± 0.300^a^	**8.3**	3.7
	Creatine-393	0.354 ± 0.010^c^	0.811 ± 0.059^b^	2.780 ± 0.211^a^	**7.9**	3.4
	Glutamate-213	0.656 ± 0.042^b^	0.841 ± 0.063^b^	2.608 ± 0.134^a^	4.0	3.1
	Glutamate-245	0.647 ± 0.035^c^	0.913 ± 0.074^b^	2.364 ± 0.103^a^	3.7	2.6
	Glycine-356	0.263 ± 0.008^b^	0.530 ± 0.042^b^	2.579 ± 0.734^a^	**9.8**	4.9
	Histidine-705	0.106 ± 0.011^b^	0.125 ± 0.010^b^	0.655 ± 0.073^a^	**6.2**	**5.2**
	Isoleucine-101	0.052 ± 0.003^b^	0.054 ± 0.005^b^	0.136 ± 0.010^a^	2.6	2.5
	Leucine-096	0.089 ± 0.006^b^	0.077 ± 0.009^b^	0.221 ± 0.019^a^	2.5	2.9
	Lysine-173	1.304 ± 0.073^b^	1.340 ± 0.130^b^	4.340 ± 0.268^a^	3.3	3.2
	Lysine-302	0.665 ± 0.045^b^	0.644 ± 0.064^b^	1.957 ± 0.147^a^	2.9	3.0
	Phenylalanine-738	0.179 ± 0.011^a^	0.019 ± 0.002^c^	0.053 ± 0.003^b^	0.3	2.8
	Tyrosine-690	0.140 ± 0.014^b^	0.201 ± 0.021^b^	0.617 ± 0.057^a^	4.4	3.1
	Tyrosine-719	0.133 ± 0.013^b^	0.178 ± 0.021^b^	0.454 ± 0.031^a^	3.4	2.6
	Valine-099	0.057 ± 0.003^b^	0.077 ± 0.008^b^	0.194 ± 0.013^a^	3.4	2.5
	Valine-104	0.059 ± 0.003^b^	0.077 ± 0.009^b^	0.178 ± 0.014^a^	3.0	2.3
Carbohydrates and conjugates	α-glucose-524	0.918 ± 0.080^a^	0.831 ± 0.148^a^	0.851 ± 0.222^a^	0.9	1.0
	β-glucose-465	1.332 ± 0.117^b^	1.399 ± 0.220^b^	2.641 ± 0.354^a^	2.0	1.9
	Glucose-341	2.901 ± 0.219^b^	2.979 ± 0.417^b^	4.840 ± 0.610^a^	1.7	1.6
	Glucose-389	0.814 ± 0.065^b^	0.841 ± 0.116^b^	1.477 ± 0.179^a^	1.8	1.8
	Glucose-391	0.645 ± 0.051^b^	0.696 ± 0.092^b^	1.231 ± 0.141^a^	1.9	1.8
	Mannose-519	0.095 ± 0.007^b^	0.259 ± 0.017^b^	0.812 ± 0.113^a^	**8.5**	3.1
Carboxylic acids and derivatives	Cis-aconitate-566	0.029 ± 0.001^b^	0.062 ± 0.004^b^	0.479 ± 0.022^a^	*16.5*	**7.7**
	Citrate-254	1.497 ± 0.068^b^	1.788 ± 0.099^b^	4.032 ± 0.340^a^	2.7	2.3
	Formate-845	0.153 ± 0.009^a^	0.197 ± 0.015^a^	0.089 ± 0.024^b^	0.6	0.5
	Fumarate-652	0.013 ± 0.001^b^	0.020 ± 0.003^a^	0.020 ± 0.002^a^	1.5	1.0
Hydroxy acids and derivatives	2-HObutyrate-090	0.055 ± 0.004^a^	0.050 ± 0.006^a^	ND		
	3-HObutyrate-120	3.678 ± 0.278^b^	7.880 ± 1.370^a^	7.906 ± 2.194^a^	2.1	1.0
	3-HObutyrate-230	1.391 ± 0.101^b^	2.984 ± 0.494^a^	3.610 ± 0.788^a^	2.6	1.2
	3-HObutyrate-241	1.665 ± 0.112^b^	3.362 ± 0.523^a^	4.441 ± 0.832^a^	2.7	1.3
	3-HObutyrate-416	1.229 ± 0.085^b^	2.656 ± 0.396^a^	3.347 ± 0.635^a^	2.7	1.3
	3-HOisobutyrate-107	0.291 ± 0.016^a^	0.418 ± 0.070^a^	0.392 ± 0.093^a^	1.3	0.9
	Lactate-411	1.301 ± 0.111^b^	0.824 ± 0.118^c^	1.996 ± 0.137^a^	1.5	2.4
Nucleosides, nucleotides, and analogues	Xanthosine-585	0.028 ± 0.001^c^	0.085 ± 0.006^b^	0.288 ± 0.015^a^	*10.3*	3.4
	Cytidine-606	0.050 ± 0.003^b^	0.087 ± 0.011^b^	0.377 ± 0.030^a^	**7.5**	4.3
	Inosine-609	0.034 ± 0.002^b^	0.045 ± 0.004^b^	0.112 ± 0.008^a^	3.3	2.5
	Inosine-823	0.033 ± 0.002^b^	0.046 ± 0.005^b^	0.101 ± 0.008^a^	3.1	2.2
	Inosine-833	0.037 ± 0.002^b^	0.067 ± 0.006^b^	0.235 ± 0.017^a^	**6.4**	3.5
Pyrimidines and derivatives	Uracil-580	0.055 ± 0.002^c^	0.156 ± 0.009^b^	0.557 ± 0.033^a^	*10.1*	3.6
	Uracil-754	0.030 ± 0.001^b^	0.063 ± 0.003^a^	ND		
Purines and derivatives	Hypoxanthine-819	0.058 ± 0.001^c^	0.124 ± 0.008^b^	0.460 ± 0.032^a^	**7.9**	3.7
Keto acids and derivatives	Pyruvate-237	0.330 ± 0.026^b^	0.267 ± 0.030^b^	0.516 ± 0.052^a^	1.6	1.9
	2-oxoisocaproate-093	0.077 ± 0.004^c^	0.221 ± 0.024^b^	0.632 ± 0.066^a^	**8.2**	2.9
	2-oxoisocaproate-095	0.052 ± 0.004^a^	0.058 ± 0.006^a^	ND		
	2-oxoisocaproate-261	0.050 ± 0.002^b^	0.060 ± 0.005^b^	0.194 ± 0.009^a^	3.9	3.2
Fatty acid esters	2-HOisovalerate-084	0.024 ± 0.001^b^	0.023 ± 0.002^b^	0.042 ± 0.002^a^	1.8	1.8
	3-methyl-2-oxovalerate-110	0.131 ± 0.008^b^	0.199 ± 0.029^b^	0.290 ± 0.045^a^	2.2	1.5
Organic nitrogen compounds	O-Phosphocholine-322	0.442 ± 0.023^b^	0.596 ± 0.049^b^	1.232 ± 0.125^a^	2.8	2.1
	Choline-320	0.401 ± 0.024^c^	0.766 ± 0.050^b^	1.768 ± 0.197^a^	4.4	2.3
Carbonyl compounds	Kynurenine-680	ND	0.181 ± 0.016^b^	1.405 ± 0.135^a^		**7.8**
	Kynurenine-686	ND	0.240 ± 0.020^b^	1.688 ± 0.174^a^		**7.0**
	Kynurenine-740	ND	0.204 ± 0.015^b^	1.415 ± 0.114^a^		**6.9**
	3-HOkynurenine-669	ND	0.050 ± 0.004^b^	0.578 ± 0.056^a^		*11.6*
	3-HOkynurenine-747	ND	0.068 ± 0.006^b^	0.689 ± 0.063^a^		*10.1*
Pyridines and derivatives	Nicotinate-825	0.013 ± 0.001	ND	ND		
	Nicotinate-861	0.010 ± 0.001	ND	ND		
	Nicotinate-894	0.009 ± 0.001	ND	ND		
Imidazoles	Urocanate-637	ND	0.008 ± 0.001^b^	0.066 ± 0.011^a^		**8.3**
	Urocanate-640	ND	0.006 ± 0.001^b^	0.060 ± 0.012^a^		*10.0*
Amines	Dimethylamine-272	0.096 ± 0.015^b^	0.454 ± 0.057^b^	2.066 ± 0.285^a^	*21.5*	4.6
Sulfones	Dimethylsulfone-315	0.215 ± 0.020^b^	0.285 ± 0.018^b^	0.707 ± 0.064^a^	3.3	2.5
Others	3-methyl-2-oxovalerate + Pantothenate-089	0.078 ± 0.004^c^	0.229 ± 0.024^b^	0.598 ± 0.052^a^	**7.7**	2.6
	Acetate + Lysine-191	1.831 ± 0.090^b^	2.048 ± 0.185^b^	6.375 ± 0.323^a^	3.5	3.1
	Lactate + Threonine-133	3.913 ± 0.361^c^	2.500 ± 0.369^b^	8.171 ± 0.452^a^	2.1	3.3
	Uridine-Cytidine-591	0.194 ± 0.015^b^	0.346 ± 0.044^b^	0.983 ± 0.111^a^	**5.1**	2.8

Values are means expressed in arbitrary units (area/TSP area) ± S.E.M. Italics and bold indicate metabolites that increase significantly between E10 and E17, and between E14 and E17 with a fold change greater than or equal to 10 and 5, respectively. Underline indicate metabolites that decrease significantly in concentration (fold change less than 1). The stage effect was analyzed by ANOVA while the means were compared with a Student’s t test (P ≤ 0.05). ^a,b,c^ Values without a common letter are significantly different. ND: not detected. The class named “others” was not considered in the identification of the individual metabolites because there are spectral areas where some molecules overlap.

A total of 37, 39 and 38 metabolites were identified in the allantoic fluid of the pHu lines at E10, E14 and E17, respectively (Tables 1 and 2). The results indicated a disappearance of nicotinate between E10 and E14 and of 2-HObutyrate between E14 and E17, while kynurenine, 3-HOkynurenine, and urocanate were detected in the allantoic fluid only at E14. Analysis of the metabolic profiles also indicated a significant increase in most metabolites during development, either between E10 and E17 or between E14 and E17. This increase was particularly pronounced for glycine in pHu−, xanthosine in pHu+, cis-aconitate, uracil, 3-HOkynurenine, urocanate and dimethylamine in both lines (fold change ≥ 10) and to a lesser extent for tyrosine and xanthosine in pHu−, betaine and glycine in pHu+, creatine, histidine, mannose, cytidine, inosine, hypoxanthine, 2-oxoisocaproate and kynurenine in both lines (fold change ≥ 5). Only formate, lactate and phenylalanine showed different profiles. Formate abundance decreased significantly between E14 and E17, while lactate (at least in pHu +) and phenylalanine abundance decreased between E10 and E14 and then increased until E17. Finally, some of the metabolites such as betaine, histidine, isoleucine, lysine, valine, glucose, etc. accumulated more rapidly in the allantoic fluid of pHu− than in pHu+ (between E10 and E14).

**Table 2:** List of metabolites identified at E10, E14 and E17 in the allantoic fluid of pHu− eggs.

Classes	Metabolites pHu−	E10	E14	E17	Fold change E17/E10	Fold change E17/E14
Amino acids, peptides, and analogues	Alanine-148	0.254 ± 0.024^b^	0.310 ± 0.022^b^	0.628 ± 0.040^a^	2.5	2.0
	Betaine-327	2.483 ± 0.151^c^	5.609 ± 0.381^b^	10.585 ± 1.447^a^	4.3	1.9
	Betaine-390	0.652 ± 0.037^c^	1.332 ± 0.085^b^	3.054 ± 0.364^a^	4.7	2.3
	Creatine-304	0.458 ± 0.016^c^	1.194 ± 0.067^b^	3.686 ± 0.467^a^	**8.0**	3.1
	Creatine-393	0.376 ± 0.014^c^	1.023 ± 0.052^b^	2.914 ± 0.322^a^	**7.8**	2.8
	Glutamate-213	0.620 ± 0.052^c^	1.252 ± 0.050^b^	2.600 ± 0.184^a^	4.2	2.1
	Glutamate-245	0.613 ± 0.050^c^	1.268 ± 0.051^b^	2.223 ± 0.154^a^	3.6	1.8
	Glycine-356	0.273 ± 0.013^b^	0.794 ± 0.038^b^	2.789 ± 1.079^a^	*10.2*	3.5
	Histidine-705	0.089 ± 0.011^c^	0.203 ± 0.021^b^	0.760 ± 0.068^a^	**8.5**	3.7
	Isoleucine-101	0.042 ± 0.004^c^	0.072 ± 0.006^b^	0.144 ± 0.014^a^	3.4	2.0
	Leucine-096	0.069 ± 0.007^b^	0.103 ± 0.011^b^	0.219 ± 0.023^a^	3.2	2.1
	Lysine-173	1.238 ± 0.104^c^	2.039 ± 0.135^b^	3.618 ± 0.180^a^	2.9	1.8
	Lysine-302	0.609 ± 0.054^c^	0.948 ± 0.074^b^	1.490 ± 0.102^a^	2.4	1.6
	Phenylalanine-738	0.175 ± 0.011^a^	0.022 ± 0.002^c^	0.059 ± 0.006^b^	0.3	2.7
	Tyrosine-690	0.115 ± 0.012^c^	0.277 ± 0.023^b^	0.663 ± 0.075^a^	**5.8**	2.4
	Tyrosine-719	0.110 ± 0.011^c^	0.240 ± 0.021^b^	0.422 ± 0.039^a^	3.8	1.8
	Valine-099	0.049 ± 0.004^c^	0.105 ± 0.008^b^	0.205 ± 0.019^a^	4.2	2.0
	Valine-104	0.052 ± 0.004^c^	0.104 ± 0.007^b^	0.185 ± 0.018^a^	3.6	1.8
Carbohydrates and conjugates	α-glucose-524	0.786 ± 0.098^b^	1.182 ± 0.148^b^	1.707 ± 0.288^a^	2.2	1.4
	β-glucose-465	1.134 ± 0.145^c^	2.003 ± 0.217^b^	4.063 ± 0.505^a^	3.6	2.0
	Glucose-341	2.563 ± 0.282^c^	4.216 ± 0.399^b^	6.854 ± 0.844^a^	2.7	1.6
	Glucose-389	0.710 ± 0.080^c^	1.163 ± 0.115^b^	2.074 ± 0.262^a^	2.9	1.8
	Glucose-391	0.569 ± 0.063^c^	0.953 ± 0.091^b^	1.713 ± 0.212^a^	3.0	1.8
	Mannose-519	0.083 ± 0.009^c^	0.316 ± 0.024^b^	0.736 ± 0.097^a^	**8.9**	2.3
Carboxylic acids and derivatives	Cis-aconitate-566	0.030 ± 0.002^c^	0.070 ± 0.004^b^	0.475 ± 0.018^a^	*15.8*	**6.8**
	Citrate-254	1.541 ± 0.057^c^	2.327 ± 0.162^b^	3.080 ± 0.308^a^	2.0	1.3
	Formate-845	0.147 ± 0.011^a^	0.125 ± 0.016^a^	0.065 ± 0.011^b^	0.4	0.5
	Fumarate-652	0.012 ± 0.001^b^	0.022 ± 0.003^a^	0.014 ± 0.002^b^	1.2	0.6
Hydroxy acids and derivatives	2-HObutyrate-090	0.046 ± 0.006^a^	0.054 ± 0.006^a^	ND		
	3-HObutyrate-120	3.392 ± 0.298^b^	7.968 ± 1.486^a^	4.043 ± 1.028^b^	1.2	0.5
	3-HObutyrate-230	1.287 ± 0.109^b^	3.071 ± 0.531^a^	2.217 ± 0.349^ab^	1.7	0.7
	3-HObutyrate-241	1.559 ± 0.127^b^	3.526 ± 0.563^a^	2.938 ± 0.420^a^	1.9	0.8
	3-HObutyrate-416	1.162 ± 0.087^b^	2.809 ± 0.438^a^	2.282 ± 0.290^a^	2.0	0.8
	3-HOisobutyrate-107	0.298 ± 0.023^b^	0.479 ± 0.076^a^	0.309 ± 0.068^ab^	1.0	0.6
	Lactate-411	1.133 ± 0.156^b^	0.977 ± 0.113^b^	1.829 ± 0.145^a^	1.6	1.9
Nucleosides, nucleotides, and analogues	Xanthosine-585	0.028 ± 0.001^c^	0.114 ± 0.004^b^	0.274 ± 0.013^a^	**9.8**	2.4
	Cytidine-606	0.040 ± 0.004^c^	0.080 ± 0.006^b^	0.288 ± 0.016^a^	**7.2**	3.6
	Inosine-609	0.035 ± 0.003^c^	0.058 ± 0.005^b^	0.093 ± 0.006^a^	2.7	1.6
	Inosine-823	0.033 ± 0.003^c^	0.058 ± 0.005^b^	0.097 ± 0.008^a^	2.9	1.7
	Inosine-833	0.038 ± 0.003^c^	0.086 ± 0.005^b^	0.228 ± 0.016^a^	**6.0**	2.7
Pyrimidines and derivatives	Uracil-580	0.055 ± 0.003^c^	0.217 ± 0.008^b^	0.550 ± 0.024^a^	*10.0*	2.5
	Uracil-754	0.034 ± 0.002^b^	0.103 ± 0.010^a^	ND		
Purines and derivatives	Hypoxanthine-819	0.069 ± 0.003^c^	0.178 ± 0.013^b^	0.519 ± 0.070^a^	**7.5**	2.9
Keto acids and derivatives	Pyruvate-237	0.285 ± 0.030^b^	0.311 ± 0.037^b^	0.454 ± 0.037^a^	1.6	1.5
	2-oxoisocaproate-093	0.070 ± 0.005^c^	0.228 ± 0.022^b^	0.544 ± 0.062^a^	**7.8**	2.4
	2-oxoisocaproate-095	0.045 ± 0.005^b^	0.067 ± 0.005^a^	ND		
	2-oxoisocaproate-261	0.044 ± 0.004^c^	0.072 ± 0.004^b^	0.202 ± 0.019^a^	4.6	2.8
Fatty acid esters	2-HOisovalerate-084	0.023 ± 0.002^b^	0.026 ± 0.002^b^	0.040 ± 0.002^a^	1.7	1.5
	3-methyl-2-oxovalerate-110	0.118 ± 0.010^b^	0.222 ± 0.032^a^	0.264 ± 0.035^a^	2.2	1.2
Organic nitrogen compounds	O-Phosphocholine-322	0.414 ± 0.021^c^	0.801 ± 0.065^b^	1.359 ± 0.141^a^	3.3	1.7
	Choline-320	0.467 ± 0.029^c^	0.967 ± 0.082^b^	1.554 ± 0.198^a^	3.3	1.6
Carbonyl compounds	Kynurenine-680	ND	0.238 ± 0.022^b^	1.671 ± 0.199^a^		**7.0**
	Kynurenine-686	ND	0.318 ± 0.027^b^	1.901 ± 0.215^a^		**6.0**
	Kynurenine-740	ND	0.272 ± 0.021^b^	1.883 ± 0.232^a^		**6.9**
	3-HOkynurenine-669	ND	0.064 ± 0.004^b^	0.648 ± 0.070^a^		*10.1*
	3-HOkynurenine-747	ND	0.090 ± 0.006^b^	0.762 ± 0.078^a^		**8.5**
Pyridines and derivatives	Nicotinate-825	0.016 ± 0.001	ND	ND		
	Nicotinate-861	0.012 ± 0.001	ND	ND		
	Nicotinate-894	0.010 ± 0.001	ND	ND		
Imidazoles	Urocanate-637	ND	0.009 ± 0.001^b^	0.105 ± 0.033^a^		*11.7*
	Urocanate-640	ND	0.007 ± 0.001^b^	0.100 ± 0.034^a^		*14.3*
Amines	Dimethylamine-272	0.070 ± 0.008^c^	0.585 ± 0.067^b^	2.684 ± 0.338^a^	*38.3*	4.6
Sulfones	Dimethylsulfone-315	0.223 ± 0.024^c^	0.342 ± 0.028^b^	0.586 ± 0.066^a^	2.6	1.7
Others	3-methyl-2-oxovalerate + Pantothenate-089	0.075 ± 0.004^c^	0.242 ± 0.025^b^	0.512 ± 0.061^a^	**6.8**	2.1
	Acetate + Lysine-191	1.816 ± 0.143^c^	3.074 ± 0.164^b^	5.637 ± 0.280^a^	3.1	1.8
	Lactate + Threonine-133	3.385 ± 0.524^b^	3.137 ± 0.336^b^	7.662 ± 0.568^a^	2.3	2.4
	Uridine-Cytidine-591	0.156 ± 0.014^c^	0.380 ± 0.028^b^	0.653 ± 0.050^a^	4.2	1.7

Values are means expressed in arbitrary units (area/TSP area) ± S.E.M. Italics and bold indicate metabolites that increase significantly between E10 and E17, and between E14 and E17 with a fold change greater than or equal to 10 and 5, respectively. Underline indicate metabolites that decrease significantly in concentration (fold change less than 1). The stage effect was analyzed by ANOVA while the means were compared with a Student’s t test (P ≤ 0.05). ^a,b,c^Values without a common letter are significantly different. ND: not detected. The class named “others” was not considered in the identification of the individual metabolites because there are spectral areas where some molecules overlap.

### Multivariate analysis of allantoic fluid spectral data

**Figure 2:**
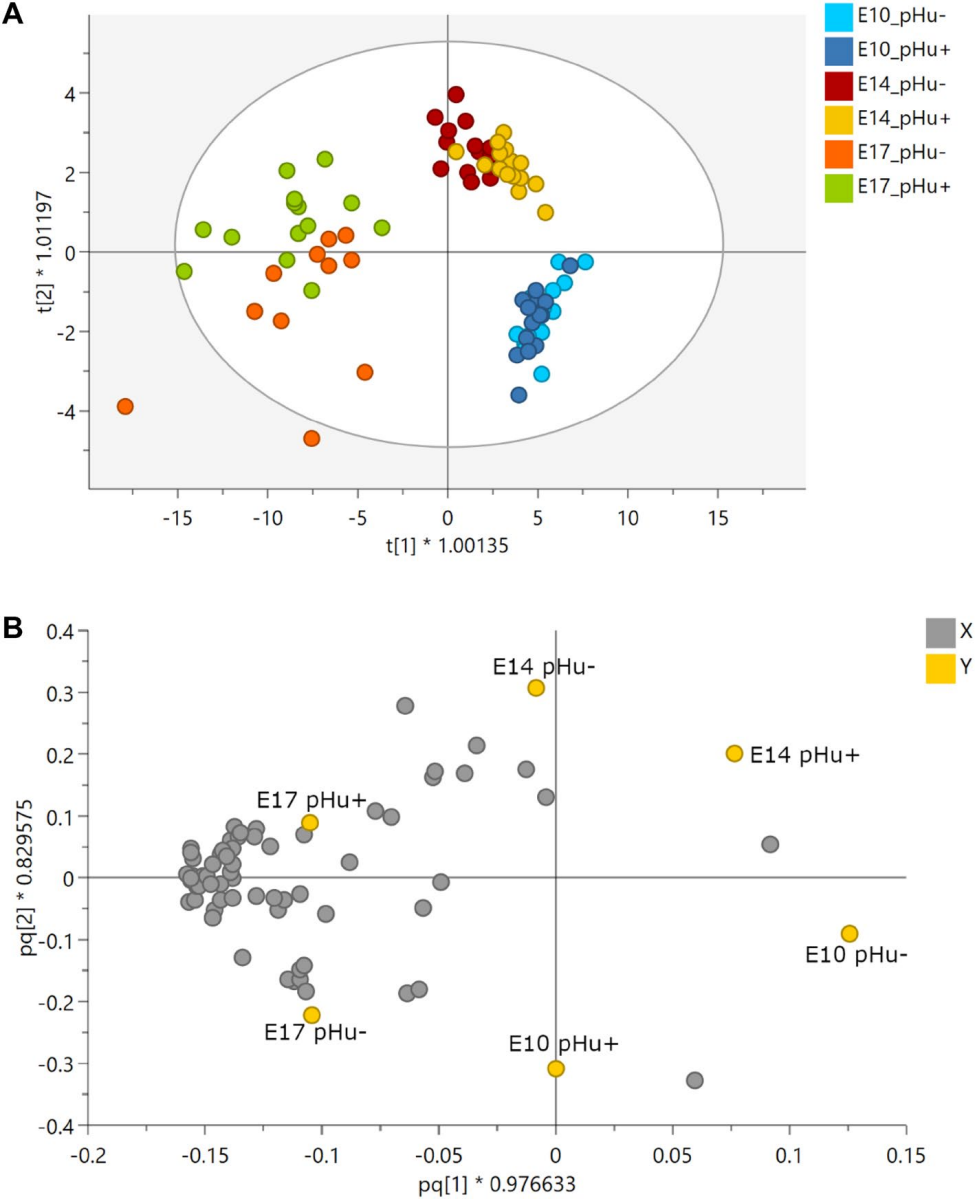
OPLS-DA score (**A**) and loading plot (**B**) based on the ^1^H-NMR spectra of allantoic fluid samples from pHu+ and pHu− lines on days 10, 14 and 17 of incubation. The OPLS-DA score plot revealed clustering of samples by embryonic stage (n = 15 per line and per stage). R^2^Y = 0.50, Q^2^ = 0.39 and CV-ANOVA = 5.6e-11. The OPLS-DA loading plot represents the correlation structure of the metabolomic variables (X, gray circle) with the stage and line variables (Y, yellow circle). The loading plot revealed that most metabolites are highly correlated with the 17th day of incubation.

The OPLS-DA performed on ^1^H-NMR spectral data was used to visualize differences in the metabolic profiles of pHu+ and pHu− lines at E10, E14 and E17 (Fig. 2A) and the distribution of the 64 variables contributing to the model (Fig. 2B). The score plot showed a clear separation between the E10, E14 and E17 stages, meaning that the stage effect was greater than the line effect. Moreover, we observed an increase in the variability of the metabolome during embryonic development. Indeed, the samples collected at E17 were more scattered compared to those of the E10 and E14 stages (Fig. 2A). In the loading plot, most metabolites were associated with the E17 stage (Fig. 2B), likely reflecting the gradual accumulation of these metabolites during embryo development (Table 1).

**Figure 3:**
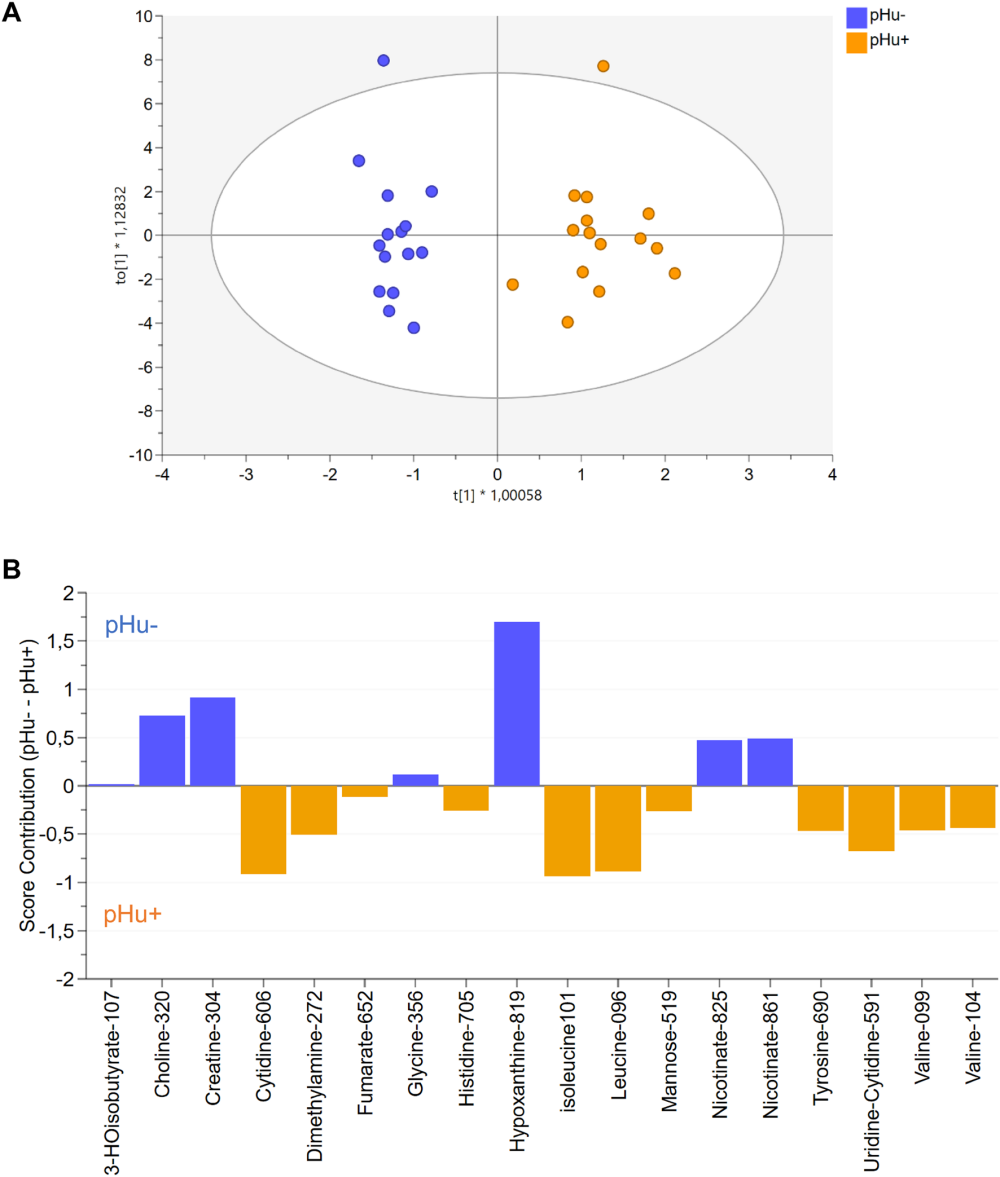
OPLS-DA score plot based on the ^1^H-NMR spectra of allantoic fluid samples from pHu+ and pHu− lines (n = 15) on the 10th day of incubation (**A**). The OPLS-DA contribution plot indicates the contribution of discriminating variables in each line. Variables with positive contributions (blue bars) correspond to metabolites more abundant in pHu− line (e.g., choline, creatine). Variables with negative contributions (orange bars) correspond to metabolites more abundant in pHu+ line (e.g., cytidine, leucine). Variables with a strong contribution such as hypoxanthine in pHu− or isoleucine in pHu+ correspond to the most discriminating metabolites between the two lines. Metabolites were referenced by their names, followed by their chemical shift multiplied by 100 (**B**). R^2^Y = 0.92, Q^2^ = 0.81, CV-ANOVA = 5.1e-05 and X = 18.

At E10, the model adjusted from the metabolome of both lines was composed of 1 predictive and 4 orthogonal components (Fig. 3A). It included 18 variables representing 16 metabolites (Fig. 3B). It explained 92% of the variation between the 2 groups (R^2^Y) and clearly discriminated the pHu+ and pHu− lines. The reliability of this model was assessed by CV-ANOVA (P = 5.1e-05) and the predictive ability Q^2^ was 0.81. The importance of the metabolites in the model and their contribution are presented in Fig. 3B. Of the 16 metabolites, 8 had an importance in the model (VIP) greater than 1 (hypoxanthine, isoleucine, leucine, cytidine, uridine-cytidine, creatine, choline and valine). Hypoxanthine was the most contributing variable in the model.

Among the 16 discriminating metabolites, we identified amino acids (glycine, tyrosine, isoleucine, leucine, valine, histidine), organic compounds (dimethylamine), vitamins (nicotinate, choline), sugars (mannose), Krebs cycle intermediates (fumarate), metabolites related to purines and pyrimidines (uridine, cytidine, hypoxanthine) and muscle metabolism (creatine). The 3-HOisobutyrate, resulting from the partial degradation of branched chain amino acids (mainly valine), was also discriminant between both lines. Choline, creatine, glycine, 3-HOisobutyrate, hypoxanthine and nicotinate were more abundant in the allantoic fluid of pHu−, while cytidine, dimethylamine, fumarate, histidine, isoleucine, leucine, mannose, tyrosine and valine were more abundant in the pHu+ line.

**Figure 4:**
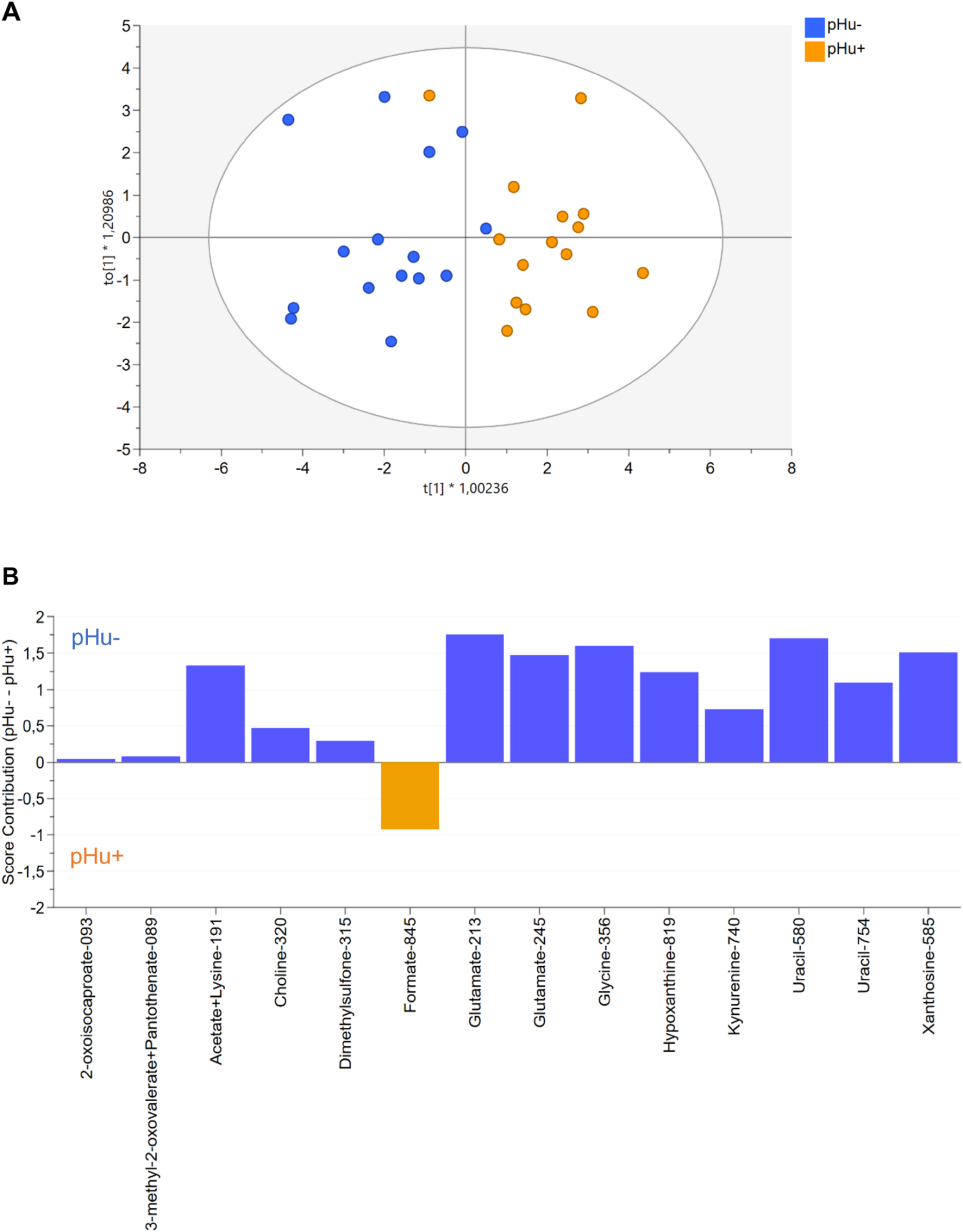
OPLS-DA score plot based on the ^1^H-NMR spectra of allantoic fluid samples 4from pHu+ and pHu− lines (n = 15) on the 14th day of incubation (**A**). OPLS-DA contribution plot indicating the contribution of discriminating variables in each line. Variables with positive contributions (blue bars) correspond to metabolites more abundant in pHu− line (e.g., hypoxanthine, uracil). Variables with negative contributions (orange bars) correspond to metabolites more abundant in pHu+ line (e.g., formate). Variables with a strong contribution such as glutamate in pHu− correspond to the most discriminating metabolites between the two lines. Metabolites were referenced by their name, followed by their chemical shift multiplied by 100 (**B**). R^2^Y = 0.68, Q^2^ = 0.56, CV-ANOVA = 0.00027 and X = 14.

At E14, the model adjusted from the metabolome of both lines was composed of 1 predictive and 1 orthogonal component (Fig. 4A). It included 14 variables representing 12 metabolites (Fig. 4B). It explained 68% of the variation between the 2 groups (R^2^Y) and clearly discriminated the pHu+ and pHu− lines. The reliability of this model was assessed by CV-ANOVA (P = 0.00027), and the predictive ability (Q^2^) was 0.56. The importance of the metabolites in the model and their contribution are presented in Fig. 4B. Of the 12 metabolites, 6 had an importance in the model (VIP) greater than 1 (glutamate, uracil, xanthosine, glycine, hypoxanthine and acetate + lysine). Formate, glutamate, glycine, hypoxanthine, uracil and xanthosine were the most contributing variables in the model.

Among the 12 discriminating metabolites, we identified amino acids and derivatives (2-oxoisocaproate, glycine, glutamate and kynurenine), organic compounds (dimethylsulfone), metabolites related to purines and pyrimidines (uracil, xanthosine and hypoxanthine), and metabolites involved in one-carbon metabolism (formate, choline). Most of them (choline, dimethylsulfone, 2-oxoisocaproate, glutamate, glycine, hypoxanthine, kynurenine, uracil and xanthosine) were overrepresented in the allantoic fluid of pHu−. Only formate was more abundant in the pHu+ line.

**Figure 5:**
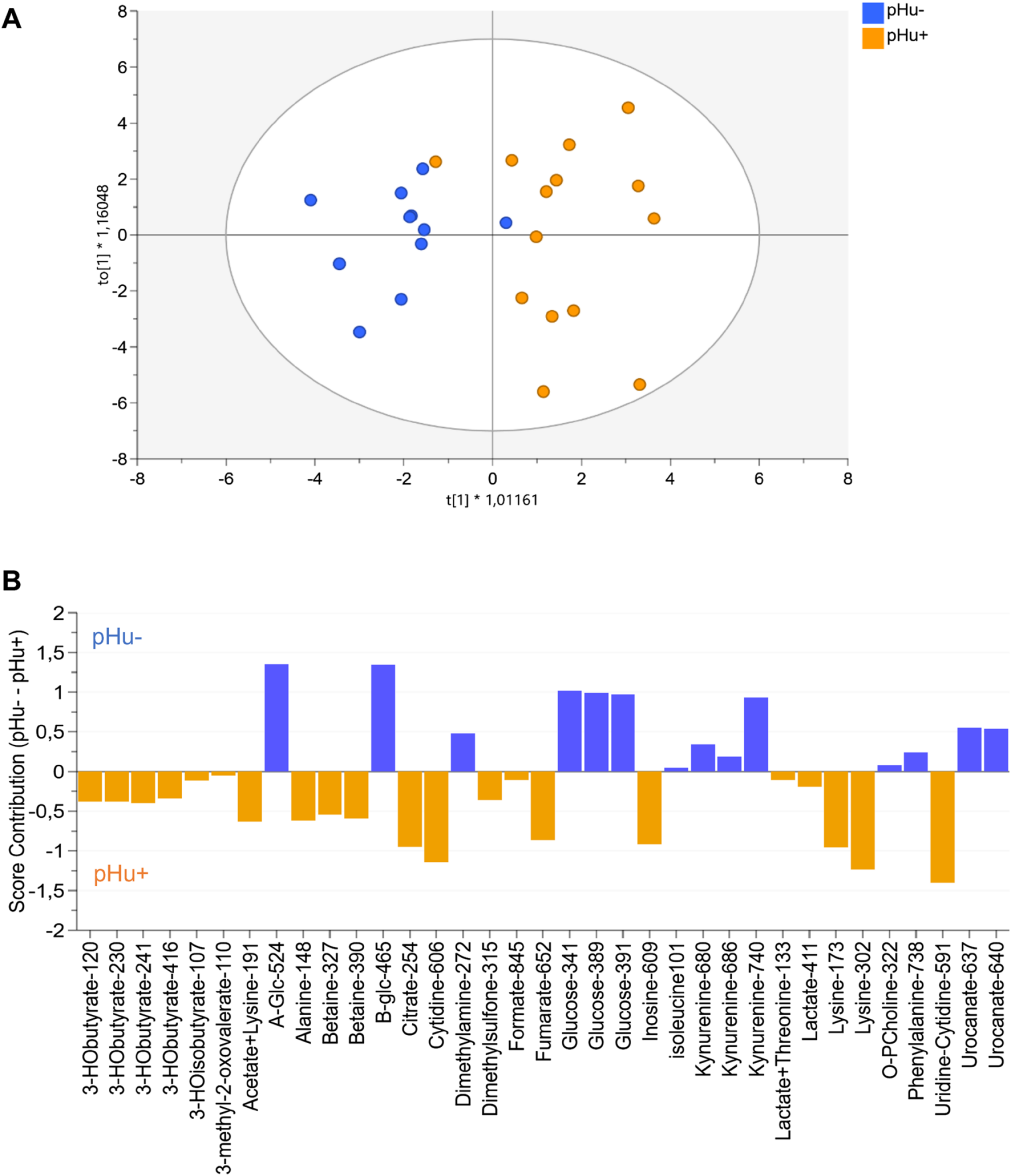
OPLS-DA score plot based on the ^1^H-NMR spectra of allantoic fluid samples from pHu+ and pHu− lines (n = 15) on the 17th day of incubation (**A**). OPLS-DA contribution plot indicating the contribution of discriminating variables in each line. Variables with positive contributions (blue bars) correspond to metabolites more abundant in pHu− line (e.g., glucose, kynurenine). Variables with negative contributions (orange bars) correspond to metabolites more abundant in pHu+ line (e.g., betaine, citrate). Variables with a strong contribution such as alpha-glucose in pHu− or lysine in pHu+ correspond to the most discriminating metabolites between the two lines. Metabolites were referenced by their name, followed by their chemical shift multiplied by 100 (**B**). R^2^Y = 0.70, Q^2^ = 0.55, CV-ANOVA = 0.0022 and X = 35.

At E17, the model adjusted from the metabolome of both lines was composed of 1 predictive and 1 orthogonal component (Fig. 5A). It included 35 variables representing 25 metabolites (Fig. 5B). It explained 70% of the variation between the 2 groups (R^2^Y) and clearly discriminated the pHu+ and pHu− lines. The reliability of this model was assessed by CV-ANOVA (P = 0.0022), and the predictive ability (Q^2^) was 0.55. The importance of the metabolites in the model and their contribution are presented in Fig. 5B. Of the 25 metabolites, 12 had an importance in the model (VIP) greater than 1 (α glucose, β glucose, glucose, uridine-cytidine, 3-HObutyrate, lysine, 3-HOisobutyrate, citrate, inosine, 3-methyl-2-oxovalerate, cytidine and kynurenine). Cytidine, glucose and lysine were the most contributing variables in the model.

Among the 25 discriminating metabolites, we identified amino acids and derivatives (3-HObutyrate, 3-HOisobutyrate, alanine, lysine, isoleucine, kynurenine, phenylalanine and urocanate), metabolites related to purines and pyrimidines (inosine, cytidine), organic compounds (3-methyl-2-oxovalerate, dimethylamine, dimethylsulfone and lactate), sugars (glucose), Krebs cycle intermediates (citrate, fumarate) and formate, a one-carbon metabolism intermediate. Betaine was considered a methyl group donor and organic osmolyte, but O-phosphocholine, an intermediate in the biosynthesis of phosphatidylcholine, was also detected. Glucose, dimethylamine, isoleucine, kynurenine, O-phosphocholine, phenylalanine and urocanate were overrepresented in the allantoic fluid of pHu−, while 3-HObutyrate, 3-HOisobutyrate, 3-methyl-2-oxovalerate, alanine, betaine, citrate, cytidine, dimethylsulfone, formate, fumarate, inosine, lactate, lysine and uridine-cytidine were overrepresented in the pHu+ line.

## Discussion

Because of the excellent reproducibility of ^1^H-NMR spectroscopy, its quantitative accuracy and its ability to identify structures^17^, this instrumental approach was of great interest to characterize the metabolome of different egg compartments such as yolk, amniotic fluid^8^ and allantoic fluid.

In birds, the composition of allantoic fluid and its evolution during embryonic development are still poorly described. The OPLS-DA performed on the spectral dataset clearly identified three clusters highlighting differences in the metabolic profiles obtained at E10, E14 and E17, regardless of the line. It also revealed an increase in inter-individual variability during embryonic development. With the exception of formate, nicotinate and phenylalanine, all metabolites accumulated in the allantoic fluid, especially amino acids, peptides and analogues, the most represented class among those identified with 12 distinct metabolites (alanine, betaine, creatine, glutamate, glycine, histidine, isoleucine, leucine, lysine, phenylalanine, tyrosine and valine). In addition to serving as building blocks for tissue protein synthesis, amino acids act as antioxidants, regulators of hormone secretion, major fuels for embryonic growth and cell signaling molecules. Amino acids are also essential precursors for the synthesis of non-protein substances with biological importance, including purine, pyrimidine nucleotides and creatine^18^. Our results are in agreement with the increase in amino acid concentration in allantoic fluid from E13 to E18 observed by Zhu et al.^19^. Changes in relative metabolic contents during *in ovo* development (e.g., amino acids) reflect significant changes in embryo metabolism, transfers of compounds between egg compartments^13^ or activation of proteases in relation to environmental acidification^10^. More generally, the accumulation mentioned above was particularly strong for some metabolites, with a fold change greater than 5. The classes mainly concerned with this high accumulation were amino acids, peptides and analogues, carbonyl compounds, and those related to purine metabolism (nucleotides, nucleosides, purines, pyrimidines).

Characterization of allantoic fluid in addition to yolk and amniotic fluid provided an overview of the nutritional environment of pHu+ and pHu− embryos^8^. Among the 40 metabolites identified in the allantoic fluid, 31 were present in the yolk and/or amniotic fluid. The 9 metabolites specifically identified in the allantoic fluid include several compounds involved in tryptophan metabolism (3-HOkynurenine, kynurenine, nicotinate), amino acid catabolism (urocanate, 3-HOisobutyrate, 2-HOisovalerate), Krebs cycle (cis-aconitate), and purine and pyrimidine metabolism (xanthosine and uracil).

Regardless of the stage (E10, E14 and E17), the OPLS-DA models based on the metabolome of the allantoic fluid of the two genetic lines divergently selected for high (pHu +) or low (pHu−) ultimate pH of the *Pectoralis major* muscle were strongly discriminant and revealed line-related differences in both energy metabolism and one-carbon and purine metabolism.

At E10, the allantoic fluid of the pHu+ contained more branched amino acids (BCAAs), such as leucine, isoleucine and valine, than that of pHu−, which was also the case for some of them in the yolk and amniotic fluid^8^. Leucine was more abundant in the egg yolk of pHu+ at E0, while leucine and isoleucine were more abundant in the amniotic fluid at E10. Valine was specifically discriminant in the allantoic fluid and only at E10.

These differences disappeared at E14 but later in development (i.e., at E17), the allantoic fluid of the pHu+ contained more 3-HObutyrate, 3-methyl-2-oxovalerate and 3-HOisobutyrate. Metabolites resulting from the catabolism of BCAAs are converted into acetyl-CoA, which acts mainly as a substrate for the Krebs cycle. As specified by Hada et al.^20^, the Krebs cycle is responsible for the complete oxidation of acetyl-CoA and the formation of intermediates required for adenosine triphosphate (ATP) production and other anabolic pathways. An overrepresentation of Krebs cycle intermediates, such as fumarate and citrate, was also observed in the allantoic fluid of the pHu+ line at E17. These results and the high concentration of alanine (the main glucogenic agent) in the pHu+ suggest a greater catabolism of amino acids in this line to produce energy, probably favored by the greater availability of BCAAs in the different extraembryonic structures (yolk, amniotic fluid and allantoic fluid). The higher abundance of 3-methyl-2-oxovalerate, 3-HObutyrate and betaine might also indicate a higher use of lipids as energy substrates in pHu+ compared to pHu− embryos. According to Zhang et al.^21^, betaine is involved in lipid metabolism by playing a role in the inhibition of fatty acid synthesis and in the stimulation of fatty acid oxidation and lipid secretion. Furthermore, 3-HObutyrate is known to act as a signal to regulate metabolism and maintain energy homeostasis during nutrient deprivation^22^. Overall, the differences observed in allantoic fluid at E17 are consistent with previous metabolomic studies performed at 6 weeks of age in serum and *Pectoralis major* muscle of pHu+ and pHu− lines, which showed that pHu+ chickens have a greater resort to lipid and amino acid catabolism, likely due to their low level of energy substrates compared to pHu−^5^.

Compared to pHu+, creatine was found in higher abundance in the allantoic fluid of pHu− at E10, similar to what was observed in amniotic fluid at the same stage^8^. Creatine is deposited by the hen in the yolk and albumen^23^ but is also synthesized de novo by the embryo^24^. It is a key compound that plays an important role in energy metabolism^25^. According to Muccini et al.^26^, creatine can maintain ATP turnover, acid–base balance and mitochondrial function. However, creatine synthesis considerably solicits arginine and methionine metabolism^27^. Approximately 50% of all S-adenosylmethionine (SAM)-derived methyl groups are consumed by guanidinoacetate methylation, the final step in creatine synthesis^28^. At E17, the allantoic fluid of pHu− contained more glucose, suggesting a greater availability of carbohydrates in the allantoic fluid of pHu− as hatching (a very energy-consuming process) approaches. The fact that the end product of anaerobic glycolysis, lactate, is less abundant in pHu− than pHu+ at this stage may indicate that aerobic recycling of lactate is less efficient in pHu+.

Nicotinate, also known as niacin or vitamin B3, was found in greater abundance in the allantoic fluid of pHu− at E10. Nicotinate is a water-soluble vitamin that plays a central role in energy metabolism and oxidative phosphorylation. In particular, it is a precursor of the bioactive molecules nicotinamide adenine dinucleotide (NAD) and nicotinamide adenine dinucleotide phosphate (NADP)^29^. In humans, nicotinate stimulates muscle mitochondrial biogenesis and respiratory chain activity^30^. Due to its anti-inflammatory, anti-oxidant and anti-apoptotic activities in a variety of cells and tissues, nicotinate is actively involved in the prevention of many pathological processes^31,32^. Nicotinate is mainly synthesized in the liver through the kynurenine pathway, which is the major catabolic route of tryptophan^33,34^. From E14 onwards, nicotinate was not detected in the allantoic fluid, while kynurenine accumulated, and to a greater extent, in the pHu− line compared to the pHu+ line.

**Figure 6:**
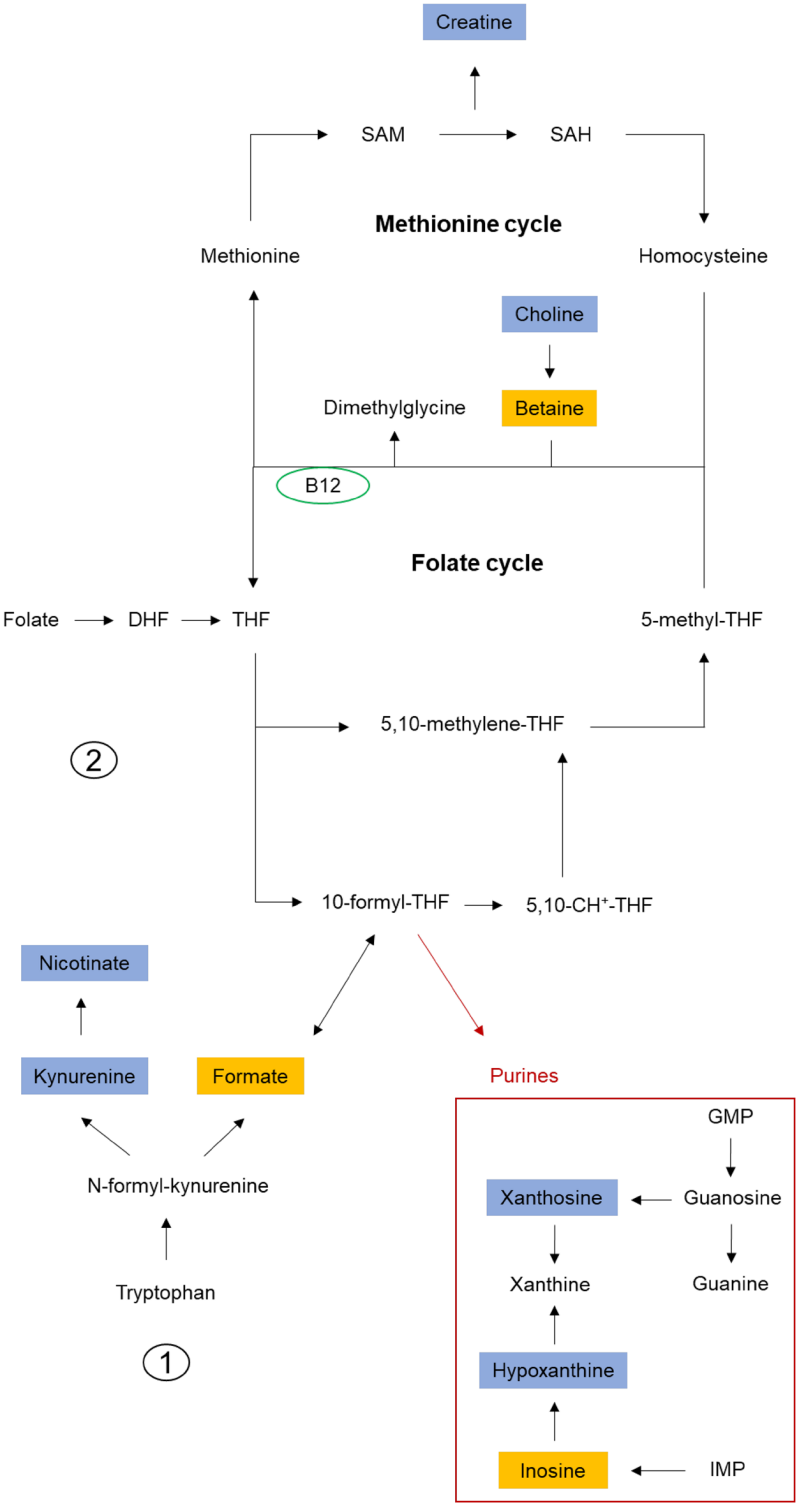
Representation of the folate and methionine cycles and the involvement of formate in one-carbon metabolism. In orange, the metabolites are more abundant in the allantoic fluid of pHu+, and in blue, the metabolites are more abundant in pHu−. **1** = Formate production through the folate-independent pathway and **2** = Formate production through the folate-dependent pathway. SAM: S-adenosylmethionine, SAH: S-adenosylhomocysteine, DHF: Dihydrofolate, THF: Tetrahydrofolate, 5,10-CH^+^-THF: 5,10-methenyl-tetrahydrofolate, B12: Vitamin B12, IMP: Inosine monophosphate, GMP: Guanosine monophosphate. Figure adapted from Brosnan and Brosnan^39^, Bozack et al.^44^, and Baroukh et al.^45^.

Through metabolome analysis of key nutrient sources for the embryo, we previously found that formate was more abundant in the yolk and amniotic fluid of the pHu− line at E10^8^. The present study showed no difference at E10 but a higher abundance of formate in pHu+ allantoic fluid at E14 and E17 compared to pHu−. At E17, betaine was also more abundant in the allantoic fluid of pHu+. Formate and betaine are both involved in one-carbon metabolism, which includes the folate and methionine cycles, as well as the trans-sulfuration pathway (Fig. 6). One-carbon metabolism is critical for early development, as it provides one carbon (1C) units for the biosynthesis of DNA, proteins and lipids and epigenetic modification of the genome^35^. Formate has been proposed as a biomarker of impaired one-carbon metabolism^36^. For instance, high levels of formate have been observed in rats deficient in vitamin B12 or folate^37^. Therefore, it is possible that the higher abundance of formate observed at E17 in the allantoic fluid of pHu+ may reflect vitamin B12 and/or folate deficiency, two metabolites playing a crucial role in the methylation cycle^38^.

Formate is produced via quite a number of processes, from different substrates, in different tissues, and in different cellular organelles. These metabolic processes occur through both folate-independent and folate-dependent reactions (Fig. 6). Regarding folate-independent reactions, formate and kynurenine can be produced from tryptophan catabolism. Interestingly, from E14 onwards, kynurenine was more abundant in the allantoic fluid of the pHu− line, while formate was more abundant in pHu+. Some formate may also be produced from histidine catabolism^39^. At E10, histidine was significantly more abundant in pHu+, while glutamate and urocanate, markers of histidine catabolism, were more abundant in the allantoic fluid of pHu− at E14 and E17. These results suggest greater histidine catabolism in the pHu− line but do not establish a direct link between histidine catabolism and formate production. However, regarding the use of formate, a significant decrease was observed between E14 and E17 in both lines. This decrease was faster in the pHu+ line than in the pHu− line with a differential of 0.072 and 0.024 arbitrary units (a.u.), respectively. This may suggest a greater utilization of this metabolite in the last third of *in ovo* development, due to greater requirements for growth and metabolism in the pHu+ line.

Several metabolites involved in purine metabolism also discriminated against the two lines. Among them, xanthosine and hypoxanthine (intermediates in guanine and adenosine metabolism, respectively) were more abundant in the allantoic fluid of the pHu− line until E14, which could explain the higher uric acid content in this line at E10 (P = 0.02, data not shown). In contrast, cytidine and inosine were more abundant in the pHu+ line at E17. Purine degradation seems to be more important in the pHu− line than in the pHu+ line, which is also characterized by a higher abundance of nucleosides (nucleic base + ribose). As de novo synthesis is very energy intensive, nucleosides could be reused through the purine recycling or rescue pathway, as evidenced by the higher abundance of inosine (hypoxanthine + ribose).

Betaine, presented above as a marker of fatty acid oxidation in the pHu+ line, is also considered a methyl group donor^40^. However, in our study, it is likely that betaine is considered more of an osmolyte, a molecule required for the maintenance of cellular integrity (protection against osmotic stress, dehydration, etc.)^41^. Indeed, a recent study showed that eggshell percentage, as well as eggshell thickness, were lower in pHu+, suggesting greater gas exchange and water loss in this line^42^. At E17, the higher betaine content in the allantoic fluid of pHu+ could therefore be a sign of a stronger adaptive response to ensure embryo protection. Other metabolites involved in embryo protection, such as glycine and glutamate, however, were more abundant in the pHu− line. These amino acids are involved in the production of glutathione, a major contributor to the redox balance in cells, through its ability to scavenge and reduce reactive oxygen species (ROS)^43^.

## Conclusions

In conclusion, the characterization of allantoic fluid by a metabolomics approach revealed different metabolic profiles during embryogenesis, as well as between the two divergent lines for their muscle glycogen reserves. At E10, pHu+ and pHu− lines have already shown a specific metabolic signature. Due to the lower availability of energy substrates, such as glucose and creatine, pHu+ embryos may use different catabolic pathways, such as amino acid catabolism and lipid oxidation, to produce energy. Finally, our study revealed that allantoic fluid could provide early and potentially accessible biomarkers of embryo metabolism.

## Methods

### Sample collection

All investigators were certified by the French government to handle animal experiments. The experiments were carried out at the PEAT INRAE Poultry Experimental Facility (2018, https://doi.org/10.15454/1.5572326250887292E12) (INRAE, Centre Val de Loire, Nouzilly, France), as described in Petit et al.^8^. The study was conducted on eggs issued from the tenth generation of the two genetic lines divergently selected for high or low breast meat ultimate pH (pHu+ and pHu−, respectively). Fertile eggs were obtained from laying hens (pHu+ and pHu−) at the peak of laying (28–29 weeks of age). Eggs were incubated under standard conditions at 37.8 °C and 56% relative humidity in the experimental hatchery of PEAT to collect the allantoic fluid on days 10 (E10), 14 (E14) and 17 (E17) of incubation. These samples were collected from the same experimental design as the yolk and amniotic fluids analyzed previously^8^. As a result, the eggs were different at each stage of sampling and egg incubation was stopped.

### Preparation of ^1^H-NMR samples

Allantoic fluids were collected from pHu+ and pHu− embryonated eggs at E10, E14 and E17 (n = 15 per line and per stage) using a syringe. The samples were centrifuged to remove cellular debris (10 min, 3000 g, 4 °C). The supernatants were ultra-filtered on Amicon columns (cut-off 3 kDa) to remove proteins and peptides of high molecular weight. The ultrafiltrates were stored at − 80 °C until NMR analysis. Ultrafiltrate samples (150 µL) were prepared by the addition of 50 µL of phosphate buffer prepared in deuterium oxide (D2O, pH = 7.4, 0.2 M) and 10 µL of trimethylsilylpropionic acid (TSP, 3.2 mM), used as an internal reference in NMR spectroscopy. Quality controls were prepared to assess analytical reproducibility by pooling 80 µL of randomly selected samples.

### ^1^H-NMR processing and analysis

The ^1^H-NMR spectra were acquired on a Bruker DRX-600 Avance III HD spectrometer (Bruker, Billerica, MA, United States) equipped with a TCI cryoprobe using a “noesypr1d” pulse sequence with a relaxation delay of 20 s, a time domain of 64 K data points, and a spectral width of 12 ppm and 64 scans. The spectra were phased, the baseline corrected and the chemical shifts referenced to the TSP. The spectra were reduced into consecutive and non-overlapping spectral regions (buckets) that were integrated and normalized by the TSP. The integration of those buckets was proportional to the concentration of the metabolites but also to the number of protons that resonate at the frequency of the peaks. Thus, each bucket represented a variable. This allowed the generation of a data matrix that was analyzed by multivariate statistics. Peak assignments were done using databases such as the Human Metabolome Database (http://www.hmdb.ca) and Chenomx NMR suite 8.1 evaluation edition (Chenomx Inc, Edmonton, Canada).

### Statistical analysis

Principal component analysis (PCA) followed by orthogonal partial least squares’ discriminant analysis (OPLS-DA) was performed using the SIMCA 16 software (version 16.0.2, Umetrics, Umea, Sweden) on the dataset. All data were scaled to unit variance. OPLS-DA is a supervised approach to discriminate groups, i.e., embryonic stage and line, using metabolomic data. Samples identified as being out of the confidence interval in the PCA (outliers) were removed from the analysis. For optimal model classification, variables with low regression coefficients and wide confidence intervals combined with low variable importance in projection (VIP) were iteratively removed. The overall quality of the models was appreciated by the proportion of variance in Y explained by the predictive component of the model (R^2^Y) and the predictive ability of the model (Q^2^). Variance analysis (CV-ANOVA) was then applied to further evaluate the significance of the results^5^. The OPLS-DA were interpreted through their score plots—a projection of the linear combinations of the metabolomics data representative of each sample on two components—and their loading plots. The loading plots represent the projection of the correlation structure of the metabolic variables (X), and the stages and lines variables (Y) on the axis pq1 et pq2 which are the combination in one vector of the loading of X weighted for the importance of the metabolites in the approximation of X (p) and the loading of Y weighted by the importance of the classes in the approximation of Y variation correlated to X (q) in the component 1 (pq1) or 2 (pq2).

In Tables 1 and 2, the main effect (stage effect) was analyzed by ANOVA while the means were compared with a Student's t test (P ≤ 0.05).

### Ethical approval and consent to participate

All animal care and experimental procedures needed for this study were conducted in accordance with current European legislation (EU Directive 2010/63/EU) and were approved by the Ethics Committee for Animal Experimentation of Val de Loire (CEEA VdL). This ethics committee is registered by the National Committee under the number C2EA 19. The use of all our live animals is respectful of animal welfare and has been thought through according to the 3Rs rule (replacement, reduction, refinement). The ARRIVE guidelines were followed while reporting the experiments on animals. The points of the guide (https://arriveguidelines.org/) have been respected. All investigators were certified by the French government to handle animal experiments.

## Data availibility

All data and spectra generated or analyzed during this study are included in the supplementary data file and have been deposited on https://zenodo.org; https://doi.org/10.5281/zenodo.7736015.

## Supplementary Information

Supplementary Information 1.

Supplementary Information 2.
