# Supplementary material for: Allantoic fluid metabolome reveals specific metabolic signatures in chicken lines different for their muscle glycogen content
Source: Sci Rep. 2023 May 31;13:8867. doi: 10.1038/s41598-023-35652-0 (PMC10232533; doi:10.1038/s41598-023-35652-0)
Supplement: Supplementary file 2 — Supplementary Information 2. [file 41598_2023_35652_MOESM2_ESM.docx]

**Supplementary Figure 1.** Representative NMR spectra of the allantoic fluid.


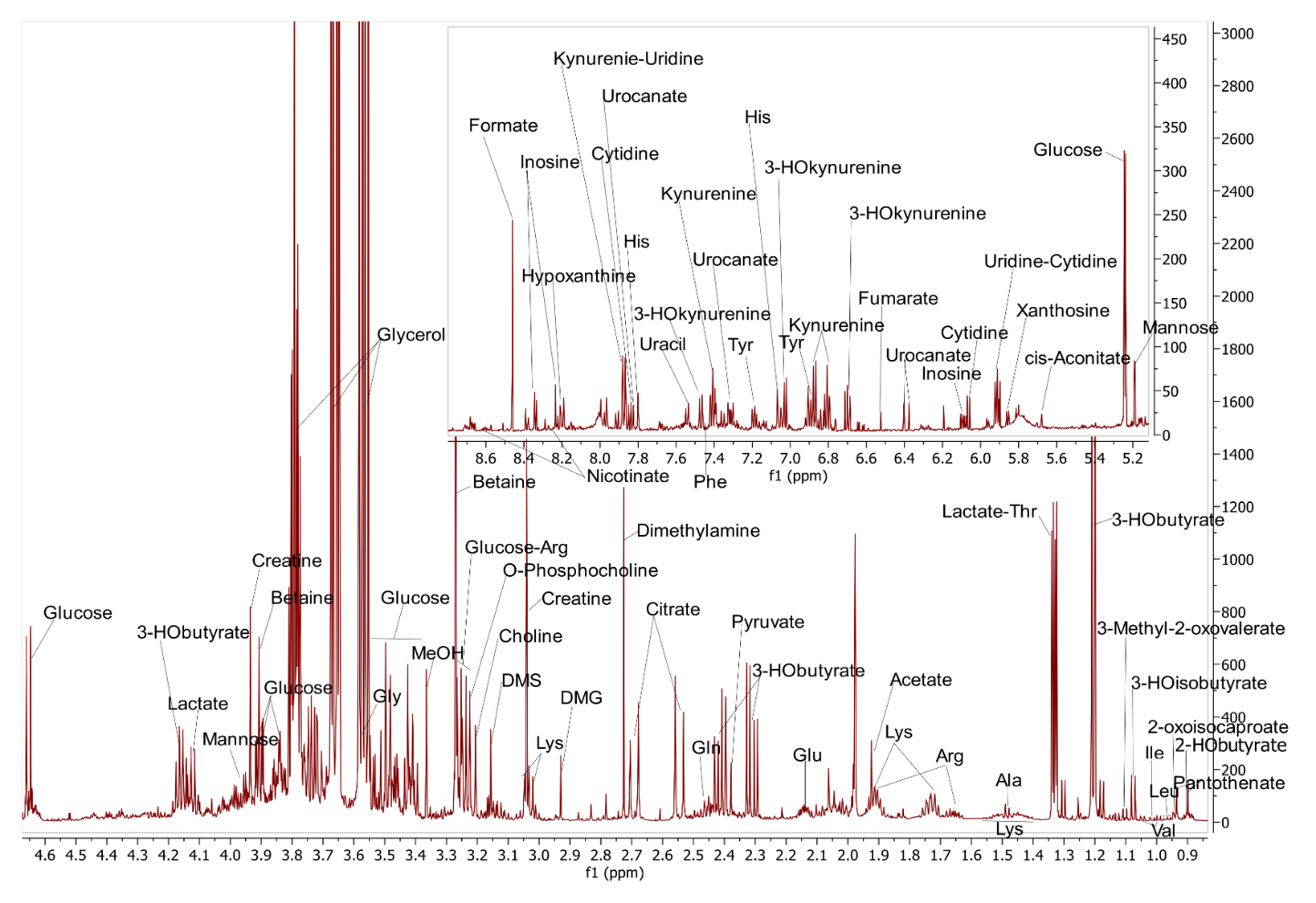


Chemical shifts are expressed in parts per million (ppm). DMS: Dimethylsulfone, DMG: Dimethylglycine, MeOH: Methanol, Tyr: Tyrosine, Gly: Glycine, Lys: Lysine, Phe: Phenylalanine, Gln: Glutamine, Glu: Glutamate, Ala: Alanine, Arg: Arginine, Leu: Leucine, Val: Valine, Ile: Isoleucine, Thr: Threonine.
